# Injectable Hydrogel versus Plastically Compressed Collagen Scaffold for Central Nervous System Applications

**DOI:** 10.1155/2018/3514019

**Published:** 2018-02-07

**Authors:** Magdalini Tsintou, Kyriakos Dalamagkas, Alexander Seifalian

**Affiliations:** ^1^Centre for Nanotechnology & Regenerative Medicine, Division of Surgery and Interventional Science, University College of London, London, UK; ^2^Nanotechnology & Regenerative Medicine Commercialisation Centre Ltd., The London BioScience Innovation Centre, London, UK

## Abstract

Central Nervous System (CNS) repair has been a challenge, due to limited CNS tissue regenerative capacity. The emerging tools that neural engineering has to offer have opened new pathways towards the discovery of novel therapeutic approaches for CNS disorders. Collagen has been a preferable material for neural tissue engineering due to its similarity to the extracellular matrix, its biocompatibility, and antigenicity. The aim was to compare properties of a plastically compressed collagen hydrogel with the ones of a promising collagen-genipin injectable hydrogel and a collagen-only hydrogel for clinical CNS therapy applications. The focus was demonstrating the effects of genipin cross-linking versus plastic compression methodology on a collagen hydrogel and the impact of each method on clinical translatability. The results showed that injectable collagen-genipin hydrogel is better clinical translation material. Full collagen compression seemed to form extremely stiff hydrogels (up to about 2300 kPa) so, according to our findings, a compression level of up to 75% should be considered for CNS applications, being in line with CNS stiffness. Taking that into consideration, partially compressed collagen 3D hydrogel systems may be a good tunable way to mimic the natural hierarchical model of the human body, potentially facilitating neural repair application.

## 1. Introduction

The limited regenerative capacity of the Central Nervous System (CNS) is what makes the neurological conditions devastating, offering poor therapeutic options to the patients. It is not only the mechanical gap that disturbs the neuronal function, but also the triggered cascade of events that leads to secondary neuronal degeneration and death. Therefore, there is a real, pressing clinical need for the development of therapeutic strategies for the currently untreatable disorders of the CNS. The advances in neural tissue engineering have provided several tools that may help in addressing those problems in the future.

### 1.1. Injectable Hydrogel Systems

Several biodegradable preformed polymeric implants have been used as drug delivery systems for sustained release, although they require invasive surgical techniques for implantation [1, 2]. Injectable hydrogels, with in situ gelling properties, provide the advantage of injection through a thin needle in a less invasive way than implantation, and, if a biodegradable polymer is used, the need for surgical removal is also eliminated.

Hydrogels have been used in 3D model technology for several years and in a wide range of tissues (e.g., bones, cartilage, and nerves) [3–7]. Hydrogels closely mimic the tissue environment because of their high water consistency and materials used, while, in parallel, their tunability makes them a kind of very flexible, highly controlled microenvironment [3, 8]. Because of the aforementioned advantages, several products are released in the market such as Matrigel [9] or Extracel [10]. All natural materials have the advantage of cell binding sites and adhesion molecules, creating a microenvironment that closely mimics the extracellular matrix (ECM) and this is why 3D models based on natural materials have attracted attention [11–13]. In this study, we chose to use collagen, which has been a material of choice for several tissue regenerative applications due to its properties.

### 1.2. Collagen Gels

Collagen-based matrices are widely used in tissue regenerative applications due to the collagen's ubiquitous presence in the human body (i.e., skin, bone, cartilage, and tendons), antigenic behaviour, and biodegradability [14–16]. Thus, it is critical to be able to utilise the mechanical properties of collagen hydrogels by cross-linking mechanisms.

However, the effectiveness of collagen-based tissue engineered materials has been severely limited by their lack of mechanical strength. A variety of methods exists to cross-link collagen gels. Physical treatments such as ultraviolet (UV) and *γ*-irradiation and dehydrothermal treatments are not practical, because of their limited use in cellular tissues. Chemical treatments with aldehydes are used to preserve and stiffen the tissues, but these treatments are highly cytotoxic. In vivo, tissues are naturally cross-linked by enzymes. However, use of these enzymes for bulk changes in mechanical properties in 3D models is not cost-effective. Chemical aldehydes are used as a fixative to preserve tissues but are highly toxic [17, 18].

### 1.3. Genipin

Genipin has been investigated to modulate mechanical stiffness of collagen and gelatin. Genipin is the active compound found in* Gardenia jasminoides* fruit extract and it cross-links collagen through cross-linking of amine groups on lysine and arginine residues, resulting in a gel strength comparable to glutaraldehyde, but it is 10,000-fold less cytotoxic [19]. In addition to an increase in mechanical strength of collagen, genipin cross-linking is associated with a colour change in which opaque collagen turns blue.

Genipin may cross-link collagen in a variety of different mechanisms. Genipin molecules may react with amino groups within a tropocollagen molecule or between adjacent tropocollagen molecules to form intrahelical and interhelical cross-links in the genipin-fixed tissue [20]. In addition, intermicrofibrillar cross-links may be formed between collagen microfibrils via polymerization of genipin molecules before cross-linking (oligomeric cross-link).

Furthermore, the degradation rate of genipin cross-linked gelatin has been found to be significantly slower than the one of glutaraldehyde-cross-linked counterparts [21]. The mechanical and rheological behaviour of genipin cross-linked gelatin has been investigated, revealing that, with an increase in genipin concentration and temperature, the gelatin network shifts from being dominated by hydrogen bonds (physical cross-links) to covalent cross-linking (chemical cross-links) [22]. Although genipin is an attractive cross-linker for collagen, its cytotoxicity at high concentrations (5 mM) limits its usage to small concentrations [23].

### 1.4. Plastic Compression

The method of plastic compression of collagen has been originally reported by Brown et al. in 2005 [24]. The method is based on the uniaxial removal of unbound water from hyper-hydrated collagen gels, reconstituted from acidic solution. As a result, collagen sheets are produced which, dependent on the application, can contain a known number of viable embedded cells.

The word “plastic” refers to the irreversible nature of the process, that is, the thickness of collagen sheets does not change (i.e., reswell) significantly in fluid once the load is removed. The main advantages of this method are simplicity, speed, and reproducibility, calculable, predictable physical, and concentration parameters, and compatibility with a viability of a resident cell population. Thus, in contrast to other techniques, the improved mechanical properties, achieved using this method, are controlled by the researcher rather than cells, but, above all, without loss of cell viability.

The advantage of multilayered compressed collagen hydrogels is that there are no progressive restrictions of the fluid leaving surface (FLS). Each new gel layer forms a new FLS when compressed. Theoretically, the thickness of each single layer is the main restriction, not their total number, as each layer is compressed individually. Also, it should be noted that this model gives an opportunity to fabricate multiple constructs simultaneously (6 to 96, depending on the well-plate format used). Additionally, by using this method, it is possible to fabricate complex multilayered tissues with different cell types or densities in each layer. It may also be possible to control cell infiltration between the layers, as it is known that increased stiffness of the matrix enhances motility of some cell types [25].

In this study, we have fabricated a promising collagen-genipin injectable hydrogel that may be friendly for CNS applications and we are comparing that to a pure collagen hydrogel and to an inherently stronger compressed collagen hydrogel in different levels of compression. Our goal is to determine the effects of genipin cross-linking compared to the effects of the plastic compression technique on the inherent properties of collagen hydrogels, as well as to check the biocompatibility of the described systems with the CNS. This will help in accomplishing the optimal in vivo functional results in later studies in order to maximise clinical potentials.

## 2. Materials and Methods

### 2.1. Injectable Collagen and Collagen-Genipin Hydrogel Fabrication

All the solutions should be kept on ice 30 minutes before the initiation of the experiment to avoid premature gelation of the collagen hydrogel solution.

The initial collagen solution was made by mixing 10% 10x Minimum Essential Medium (MEM) and 70% of rat tail collagen 2 mg/ml (Type I) (FirstLink, UK) solution in a wide base flask. This solution was then neutralised with 1 M NaOH until the colour got stabilised to a bright fuchsia (pink) colour (changed from yellow). Gelation can incur prematurely at this stage so the remaining steps of the protocol should be done very fast. The 10% cross-linking genipin diluted in phosphate-buffered saline (PBS) solutions was added (0.5 mM concentration according to the literature review) to our samples, but PBS alone was added in the control collagen-only gels (10%). PBS was added again (10%) after that step. After swirling and mixing the solution, the solutions were either kept on ice for rheometry or put in the incubator at 37°C in a 12-well plate. 3 ml was the quantity chosen for the collagen hydrogels in the 12-well plates, according to the original paper directions. All materials were purchased from Sigma-Aldrich unless otherwise stated.

All the values reported in the following sections of the paper are the averages of at least three samples for each hydrogel type and/or compression level.

### 2.2. Rheology

The mechanical properties of the hydrogels were tested through rheometry in the oscillatory mode. To get the gelation point of the hydrogel the temperature of the rheometer's plate was set to 4°C to avoid premature gelation that could alter the results.

Shear Pa was set to 0.1 and frequency to 1 rad/sec, while the temperature was set up to give some measurements for the stabilized solution at 4°C (10 seconds) then rise fast to 37°C obtaining measurements for 10 seconds and then get stabilized at 37°C (body temperature) until the storage modulus becomes equal to the loss modulus, signifying the gelation point. Mineral oil or silicon oil was applied around the hydrogel solution on the rheometry plate to avoid evaporation and questionable results. When storage and loss moduli plateaued, the test got aborted since gelation had already taken place. Using the same gelled hydrogel, the preshear oscillation with frequency sweep (0.1 rad/sec–50 rad/sec) was tested at 37°C for Shear Stress 1 Pa and frequency 1 rad/sec to get some insight on the hydrogels mechanical properties. In an attempt to simulate the room temperature versus body temperature, we also tested the same properties raising the temperature to 25°C (room temperature) instead of 37°C. Gel point was thought to be the time at which the shear storage modulus *G*′ = the shear loss modulus *G*′′.

Young's modulus was determined by calculating the slope of the steepest region of the stress-strain curve.

The expression in mathematical terms to calculate Young's modulus is the following:

(1) $$\begin{array}{c} E=\frac{\text{Stress}}{\text{Strain}}=\frac{F}{A}*\frac{L}{\Delta L}, \end{array}$$

where *F* is the force applied on the sample, *A* is the unstressed cross-sectional area through which the force is applied, *L* is the unstressed length, and Δ*L* is the change in length.

### 2.3. Degradation Assays

After the hydrogels were left in the incubator to “mature” at 37°C for either 30 minutes or 24 hours the initial weight of the gels was measured. Artificial cerebrospinal fluid (aCSF)-0.1% collagenase mixture was made and was added on the top of those gels. The solution of aCSF-0.1% collagenase was replaced every 30 minutes for the next 4 hours. For control gels, only PBS was added on the top of the gels instead of the aCSF-0.1% collagenase mixture. At the end, the collagenase solution or the PBS for the controls was removed and the wells were washed with PBS solution and were put on a shaker table for 20 minutes. The remaining partially digested gels were collected and weighed to obtain the final wet weight. The samples were freeze-dried to obtain the dry weight as well. The calculations were based on the following:

(2) $$\begin{array}{c} \text{Gel Remaining}\left(\;\%\;\right)=\frac{\left(\text{Final wet weight}\right)}{\left(\text{Initial wet weight}\right)}*100. \end{array}$$

### 2.4. Swelling Ratio

The hydrogels were incubated for 24 hours in a 12-well plate. Swelling with PBS for further 24 hours followed. Disks were scooped with tweezers and weighed immediately afterwards to obtain the swollen weight of the gels. Then, after lyophilization, the dry weight was measured and the swelling ratio [26] was calculated according to the following equation:

(3) $$\begin{array}{c} \text{Swelling ratio}=\frac{\text{Swollen weight}}{\text{Dry weight}}. \end{array}$$

### 2.5. Compressed Collagen Method

To compress the collagen hydrogels small chromatography rolls of the same diameter as the diameter of the well plate were applied on the top of each gelled hydrogel and left to be compressed.

To fabricate a 3D model, after the compression, a new collagen gel solution was added on the top of the compressed gel and it was then compressed again along with the previously compressed one. We could have added as many layers as we pleased but, for our purpose, two layers were considered to be enough, given that this study is a preliminary study focusing on CNS applications that do not require denser constructs [27]. An alternative method would have been to have one gel on the top of the other in the well and compress them all together at the end, but this would be limited by the height of the well.

### 2.6. Measurement of Fluid Loss and Time of Compression

Absorbent paper rolls were weighed on the electronic balance to two decimal points every minute for the first 5 minutes after compression and then 5 minutes until no measurable change of weight was noted. For example, if after 10 minutes of compression the weight of the paper roll did not change compared to the previous reading, which was measured after 5 minutes, time point of 5 minutes was taken as the time of full compression.

Weight gain in the absorbent paper rolls was recorded as fluid loss from the collagen gel during compression. The weight of the water removed from the gels was calculated according to the equation below:

(4) $$\begin{array}{c} \Delta W=Weight\;\;t_{n}–\text{Weight }t_{0}, \end{array}$$

where Weight *t*_*n*_ is the weight at time point *n* and Weight *t*_0_ is initial weight of paper roll (4.4 ± 0.3 g). The rate of fluid loss from the gels of different heights was calculated as

(5) $$\begin{array}{c} Q=\frac{\text{Weight }t_{n+1}-\text{Weight }t_{n}}{{Time}_{n+1}-{Time}_{n}}, \end{array}$$

where *Q* is the rate of fluid loss, weight *t*_*n*+1_ is the weight of the paper roll at the time point *n* + 1, weight *t*_*n*_ is the weight of the paper roll at the time point *n*, time_*n*+1_ is the time point *n* + 1, and time_*n*_ is the time point *n*.

### 2.7. Hydraulic Resistance of the Fluid Leaving Surface (FLS)

The hydraulic resistance of the FLS (RFLS) has been thought to be increased during the plastic compression process. *R*_FLS_ was calculated according to the following equation:

(6) $$\begin{array}{c} R_{FLS}=\frac{A\times P}{\mu \times Q}, \end{array}$$

where *R*_FLS_ is the hydraulic resistance of fluid leaving surface (FLS), *Q* is the rate of flow (in ml/min), *A* is surface area (in cm^2^), *P* is pressure over the surface (in N), and *μ* is the dynamic viscosity of water (1.002 × 10^−3^ Ns/m^2^).

### 2.8. Correlation of Compression Level to Rheology

The stiffness of the compressed collagen hydrogel was estimated according to Young's modulus measurements of the rheometer for the different % levels of plastic compression, namely, for 50, 75, and 99% collagen compression, as described in Section 2.2. for “Rheology.”

## 3. Results

### 3.1. Mechanical and Gelation Studies for Injectable Collagen Hydrogels

#### 3.1.1. Rheometry

**Figure 1:**
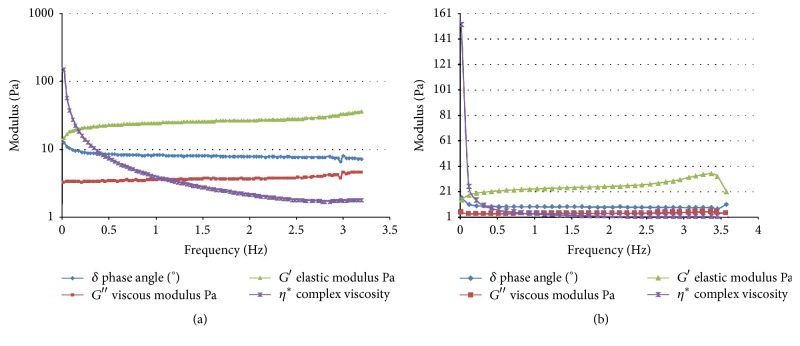
Rheometry frequency sweep for gelled collagen-genipin hydrogels in oscillatory mode (a) and gelled collagen hydrogels in oscillatory mode (b). It is illustrated that *δ* drops, so those hydrogels are viscoelastic materials. *G*′ > *G*′′ so this regards a well-structured (gelled) system. We can also see that *G*′ and *G*′′ are almost independent of frequency so, in general, sedimentation is unlikely to occur and particles are strongly associated. *G*′ of collagen-genipin hydrogels > *G*′ of collagen hydrogels and *G*′′ of collagen-genipin hydrogels < *G*′′ of collagen hydrogels.

A typical example of the rheological data at 37°C is shown in Figure 1. The gel point for the genipin cross-linked hydrogel at 37°C was on average approximately 38 seconds, whereas for the non-cross-linked collagen hydrogel it was 42 seconds. At 25°C, genipin cross-linked hydrogels gelled in 86 seconds and non-cross-linked hydrogels in 81 seconds. The fact that the genipin cross-linked hydrogels required less time to gel at 37°C compared to 25°C might be due to the unique properties of genipin and the effect of temperature in the gelation time and compressive strength, as described before [28], even though those results need to be validated with future studies. *G*′ for the collagen gels cross-linked with 0.5 mM genipin was higher than *G*′ for the collagen hydrogel and *G*′′ for cross-linked hydrogels was lower than *G*′′ for the collagen hydrogels; the results were not statistically significant though for either condition (*p* = 0.17 and *p* = 0.09, resp., for 37°C and *p* = 0.21 and *p* = 0.12). Young's moduli were ranging from 14.98 kPa up to 22.46 kPa for the first minutes after gelation both for collagen and collagen-genipin hydrogels, which is within the “CNS-friendly” range of Young modulus.

Nevertheless, it seemed like the cross-linked hydrogels kept increasing their modulus over time. After the gels were left to mature for 24 hours, rheology measurements suggested that Young's modulus significantly increased, reaching up to 110 kPa ± 21 (*p* < 0.05) for collagen-genipin hydrogels and up to 65 kPa ± 11 for collagen hydrogels (*p* < 0.05). Thus, approximately a 6-fold increase was observed in the collagen-genipin hydrogels modulus. A 48- or 72-hour testing of the mechanical properties through rheology might be useful in the future, in order to establish how long it takes for *G*′ to be saturated in collagen-genipin hydrogels, indicating the end of cross-linking. This would give us the final Young's modulus for the fully cross-linked collagen-genipin hydrogel to allow a more accurate understanding of the mechanical interaction of the hydrogel system with the CNS.

#### 3.1.2. Hydrogel Degradation Studies

**Figure 2:**
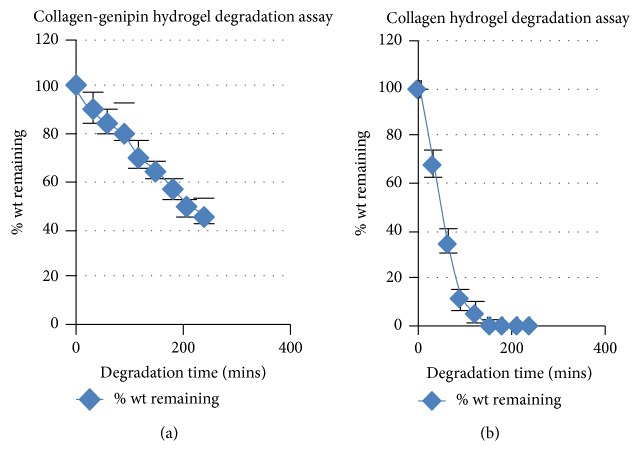
On (a), the results of the collagen-genipin hydrogel results are depicted showing a gradual decline in the % weight remaining of the hydrogel over the time due to the exposure to the 0.1% collagenase solution. On (b), the results of the same assay for a collagen hydrogel are depicted, indicating a much more rapid decline in the % weight remaining. It is noted that all gels were left in the incubator to “mature” at 37°C for 24 hours before the initiation of the assay.

The hydrogels were left to “mature” in the incubator at 37°C for 24 hours before the initiation of the degradation assay, to allow for the mechanical properties of the hydrogels to stabilize after the cross-linking with genipin. The degradation assay lasted for 4 hours and every 30 minutes measurements were conducted to check the resistance of the gels to degradation induced by the collagenase solution. PBS was used in place of the collagenase 0.1% solution for our control hydrogels. There was no difference in the weights measured during this time period for the control groups.  Figure 2 illustrates the % weight remaining after 4 hours of exposure to 0.1% collagenase for both collagen and collagen-genipin hydrogels. The results suggest that the cross-linking with genipin significantly increases the resistance of the hydrogels to the collagenase-induced degradation (*p* < 0.05).

**Figure 3:**
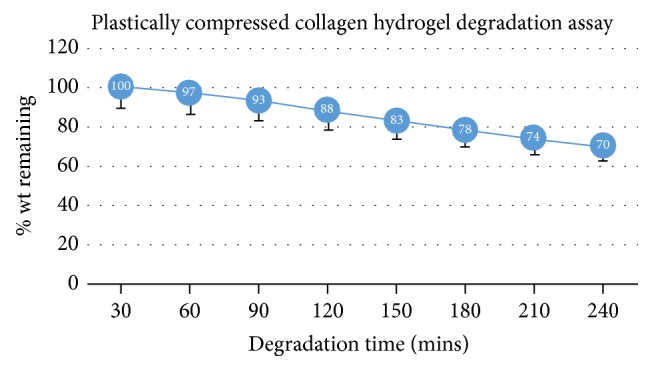
This graph depicts the degradation assay results for the plastically compressed collagen hydrogels. There is a marked resistance to the 0.1% collagenase solution compared to the other hydrogels tested.

Similarly, the degradation rate for our plastically compressed collagen hydrogels was tested. The results for the fully compressed hydrogels are shown in Figure 3. It is evident that plastic compression of the collage hydrogel significantly increased the resistance to the collagenase-induced degradation.

*Swelling Ratio*. The swelling ratios of the hydrogels were calculated according to (3) previously described, after 24-hour incubation in PBS solution. The swelling ratio of the collagen gels was 82.1 ± 2.3 and the swelling ratio of collagen-genipin gels was 103 ± 4.5. On the other hand, the swelling ratio of the fully compressed collagen hydrogels was between the two aforementioned values, but not significantly higher than the one of the uncompressed collagen hydrogels. In particular, the swelling ratio for the fully compressed collagen hydrogels was 89.4 ± 3.1.

### 3.2. Mechanical and Gelation Studies for the Compressed Collagen Hydrogels

Gels were fabricated in accordance with the aforementioned protocols and were left in the incubator at 37°C for 24 hours to “mature.” Due to the observed rheology measurements that we mentioned above, suggesting that during the first 24 hours the stiffness of collagen-genipin hydrogels significantly increases, approaching Young's modulus values of around 110 kPa, collagen-genipin hydrogels were not considered appropriate for testing through plastic compression, after taking into account our results from the compression of the collagen-only hydrogels. Besides, the primary purpose of this experiment was to compare the impact of the cross-linking of collagen with genipin to the mechanical effect of the plastic compression of collagen, as means of strengthening the inherently weak collagen hydrogels for CNS applications.

#### 3.2.1. Measurement of Fluid Loss and Time of Compression

It is known that >90% of fluid will be extracted from the hydrogels with the method of plastic compression. Using absorbent paper rolls as described before, we performed sequential weight measurements to quantify the fluid loss over time and find the compression rates of our hydrogels until the gels get fully compressed.

According to (4) mentioned above, the weight of the water that was removed from the hydrogels was calculated. The initial weight of the paper rolls was found to be around (4.32 ± 0.24 g). For the 3 ml collagen-only plastically compressed hydrogels, the initial gel height in the moulds with constant surface areas of 379.9 mm^2^ was 7.9 mm. Approximately 10 minutes were needed in order to reach full compression of those hydrogels. 92.5 ± 1.4 was the calculated percentage of total fluid loss (±SD), while Young's modulus was found to reach extremely high values after full compression (2238 ± 776 kPa).

The Young modulus was also tested for intermediate levels of fluid losses from the hydrogels in order to get a better understanding on how to tune the compression level in accordance with the desired biomedical application.  Table 1 summarises the findings.

It is noted that the difference of % fluid loss was statistically significant for the time periods 0-1 minutes and 3-4 minutes (*p* < 0.05).

#### 3.2.2. Rate of Fluid Loss

**Figure 4:**
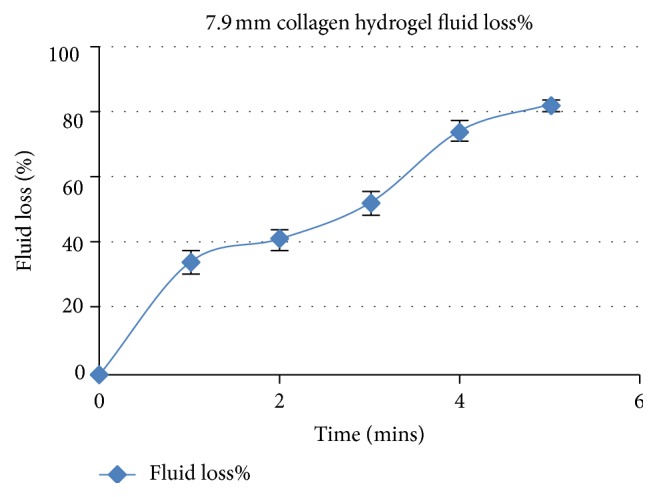
Illustration of the % fluid loss over time for collagen-only hydrogels (7.9 mm, 3 ml) for five minutes. Results are depicted as average ± SD.

The rate of fluid loss was calculated according to (5). The rate of fluid loss for the period 0-1 minutes was obviously significantly higher in comparison to all the other time points examined (1.24 ± 0.27 ml/min, *p* < 0.05) (Figure 4). For the 1-2-minute time period, the rate of fluid loss dropped significantly to 0.37 ± 0.18 ml/min (*p* < 0.05). Next, the rate of the fluid loss dropped further for the time period 2-3 minutes (0.31 ± 0.09 ml/min, *p* > 0.05). There was a statistically significant reduction of the rate of fluid loss for the remaining time periods (3-4 minutes and 4-5 minutes) in comparison to the period 2-3 minutes. In specific, the rate of fluid loss was 0.21 ± 0.019 for the time period 3-4 minutes and 0.19 ± 0.018 ml/min for the period 4-5 minutes.

#### 3.2.3. Measurement of the Hydraulic Resistance of FLS (*R*_FLS_)

It has already been accepted that the FLS of the compressed hydrogel does not have the same properties as the surface opposite to FLS. Collagen fibrils are being accumulated at the FLS site since they keep being moved during the plastic compression process towards the fluid exit point.

Utilising equation (6) mentioned above, it was found that *R*_FLS_ increased exponentially with time during compression. After 1 minute of plastic compression RFLS was 7.9 × 10^4^ ± 1.1 × 10^4^ cm^−1^, while during the 2nd minute the RFLS significantly increased to 10.04 × 10^4^ ± 0.6 × 10^4^ cm^−1^ (*p* < 0.05). The 3rd minute of compression led to another significant increase of *R*_FLS_ (13.29 × 10^4^ ± 0.9 × 10^4^ cm^−1^). The last two minutes resulted in *R*_FLS_ of 17.4 × 10^4^ ± 1.1 × 10^4^ cm^−1^ (4th minute) and 18.87 × 10^4^ ± 0.53 × 10^4^ cm^−1^ (5th minute).

### 3.3. Mechanical and Gelation Studies for Compressed Collagen 3D Hydrogel Models

#### 3.3.1. Effect of Multilayering on Dynamics of Fluid Loss from Collagen Gels during Plastic Compression

**Figure 5:**
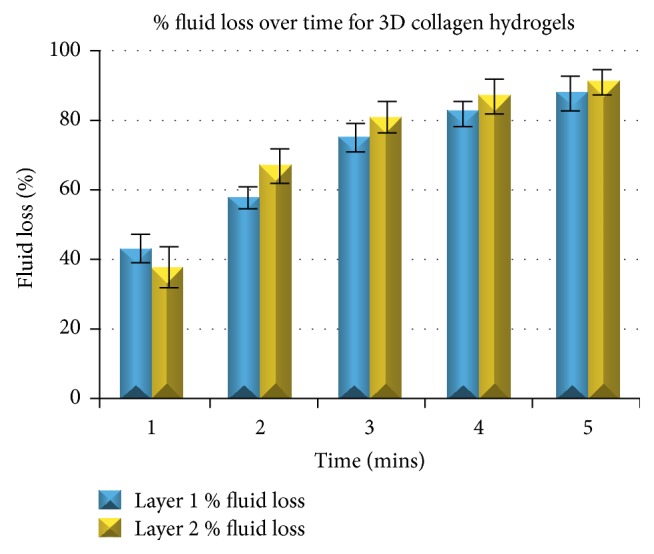
% fluid loss of both collagen layers over time for the 3D compressed collagen hydrogel model. It is noticeable that % fluid loss is always higher for the 2nd layer after the 1st minute of compression.

With the same process as described above for the single collagen hydrogels, the % of fluid loss was calculated over a 5-minute period of time until no change was observed in the weight of the paper roll, indicating full compression. The methodology for developing multilayered plastically compressed collagen 3D hydrogel models has already been described before in Section 2.5.  Figure 5 summarises our findings.

## 4. Discussion

### 4.1. Collagen and Collagen-Genipin Hydrogels

After neutralising acid solubilized collagen solution with NaOH, a modelling material is developed, which is about to gel when the temperature rises above 4°C, to form a fibrillar gel network [29]. When the collagen mixture is exposed to body temperature (37°C), the reaction kinetics have been found to occur after some seconds up to minutes [30]. Collagen has about 90 amine groups in every collagen molecule, which act as cross-linking sites for genipin and are dispersed throughout the triple helical and telopeptide regions of collagen [31].

The initial reaction of the primary amine groups with genipin triggers the formation of a second activated form of genipin, which, in turn, induces the further polymerization of genipin molecules [32].

The high resistance of the collagen-genipin hydrogels to collagenase degradation is justified due to the wide variety of intrahelical, interhelical, and intermicrofibrillar covalent cross-links throughout the collagen hydrogel [33].

It has been suggested that the impact of the genipin cross-linking on the hydrogel systems is concentration-sensitive. Based on our data the use of this genipin concentration in the described collagen hydrogel might lead to improved biocompatibility and cell viability, given the higher swelling ratio reported when compared to the collagen gels after 24 hours. This observation may be attributed to the increased mechanical strength of the gel, which allowed it to stand under its own weight and entrap water as opposed to collagen hydrogels, which sagged under their own weight. Further studies would be helpful to verify that hypothesis.

The gel point where *G*′ = *G*′′ was found to be slightly reduced for collagen gels that were cross-linked with genipin at 37°C. Thus, genipin seemed to accelerate the collagen solution gelation, even though this was not a statistically significant result.

A significant change in the shear storage modulus (*G*′) though was noted, which results in approximately 50% stiffer gels about half an hour after the cross-linking with genipin, in comparison with the collagen-only hydrogels which maintain similar moduli.

The mechanical properties of the hydrogel, including Young's modulus of the gel, are highly important for the biomedical application since it has been known that the mechanical strength of the material can affect cell growth/differentiation. Therefore, choosing the right material with the appropriate mechanical properties in accordance with the suggested application is the first step for a successful approach. The optimal material shall have an elastic modulus matching the one of the native growth environment, meaning that for our purpose we need to use a system with mechanical properties matching the ones of the spinal cord/brain tissue [34, 35].

Nevertheless, the elastic modulus of the CNS tissue is not clear, given that there is some disagreement among different studies. In general, the typical values which are mentioned lie within the range of ~3 kPa–300 kPa for spinal cord tissue [36] and ~500 Pa for brain tissue [35]. Given the soft nature of the spinal cord and of the CNS tissue in general, hydrogels are considered ideal candidates for therapeutic approaches in CNS due to the highly swollen and weak nature that matches the environment.

The moduli of all of our gels lie within the aforementioned range, showing promise for the hydrogels application in the CNS. It is noted though that *G*′ was found to gradually increase during the first 24 hours. Even though it was beyond the scope of this study to investigate that further, future work might need to reach up to the point that *G*′ gets saturated. It is definitely an interesting preliminary finding though, suggesting that collagen-genipin hydrogels need some time to “mature,” since cross-linking is still taking place, altering the mechanical properties of the hydrogels several hours after the gels' formation. On the contrary, *G*′ of collagen-only hydrogels has been found to get saturated after less than an hour. Despite that they still remain within the modulus of the CNS tissue.

Degradation experiments over a 4-hour period of time were conducted on gels matured for 24 hours to establish a kinetic profile for gel degradation. Significant increases in gel strength were obtained with 0.5 mM genipin when compared to collagen-only hydrogels. The degradation resistance increased with genipin concentration due to the increased rate of reaction at higher concentrations as analysed above, even though the resistance level was lower than the one depicted in our plastically compressed collagen hydrogels.

Overall, it is concluded that genipin shows a significant effect on the degradation properties of the hydrogel, in comparison to the weak impact on the collagen gel's mechanical properties. Taking into account that the stiffness of the gel is mainly dependent on the short range cross-links, which help in opposing the collagen fibres' motion, it is hypothesized that the existence of a wide range of short and long cross-links throughout the collagen gel induces a more significant impact on the enzyme degradation resistance of the hydrogel in comparison to the impact on the stiffness. Future work might benefit from prolonging the degradation experiment in order to validate the results of this preliminary study.

In terms of the potential cytotoxicity of genipin, this seems to be a concentration-dependent issue. Even though there are studies suggesting that cell viability can be affected when genipin is the cross-linker, potentially due to the cross-linking of the non-cross-linked genipin to the amine acids of the medium [37], tuning the genipin concentration or proceeding with frequent change of the medium, collagen-genipin hydrogels can overcome that limitation. The collagen-genipin hydrogel seems to be promising for future neural engineering applications and might enable minimally invasive therapeutic techniques in the future, but in depth in vitro and in vivo studies need to be conducted first to verify these preliminary findings.

### 4.2. Compressed Collagen Hydrogels

Collagen compressed hydrogels were developed according to the novel technique of Professor Brown [24]. The concept of the plastic compression process regards the uniaxial rapid expulsion of more than 98% interstitial fluid from the collagen hydrogels under load. Fluid loss and collagen compressed construct thickness were in agreement with the results of the original paper.

It is of great importance that, among all the tested hydrogels, the plastically compressed collagen hydrogels were the ones that demonstrated even more raised levels of resistance, significantly affecting the degradation of the plastically compressed collagen hydrogels.

The full compression of the collagen hydrogels has been previously proven not to significantly affect cell viability [38] but, according to our findings, Young's modulus has been found to increase drastically up to about 2300 kPa which goes far beyond Young's modulus value of the CNS. According to our findings, a compression level of up to 75% would be in line with the stiffness of the CNS (see Table 1); this would facilitate the application of such a hydrogel for CNS tissue engineering applications.

What is of great importance though and should be taken into account is that after the reexposure of the compressed hydrogel to fluids (e.g., PBS), it will reswell, without reaching its initial hydration status. This would potentially decrease Young's modulus but, even though this was not tested in this experiment, it is hypothesised that the compressed collagen stiffness was so high that any change would not be adequate to approach the low stiffness values of the CNS tissue. Besides, the swelling ratio was still found to be comparable to the one of the uncompressed collagen-only hydrogels, supporting our latter hypothesis. In depth analysis though might be worth being conducted in future studies.

### 4.3. Compressed-Collagen 3D Hydrogels Systems

The human organism is a hierarchical system where each tissue is a result of the assembly of many separate layers with specialised residing cells. The ultimate goal of biomedical engineering is the mimicking of such a hierarchical model, trying to assimilate the natural environment of the body to optimize the therapeutic effects. Several techniques have been developed (both cell- and biomaterial-based), but all of them have limitations and complexities that discourage their wide usage to fabricate a functional 3D model.

The method of plastic compression that is used here is easy to use, while it can be tuned to match the stiffness and layers of the natural body tissues. The interlayer connection facilitates the biomedical engineering applications, using natural polymers and vivid cells in situ.

The injectable hydrogels, on the other hand, are also 3D models, which are even friendlier for the CNS tissue; they are easy to make and can be highly tunable in order to match the needed properties to optimize our results. In addition, they do not require an open surgery, so neurosurgeons favour their use due to the less invasive nature, which could potentially lack unnecessary complications. Further tests though need to be conducted in order to establish the better therapeutic approach for CNS-related conditions.

## 5. Conclusion

To conclude, the method of collagen plastic compression is a very promising and easy to use technique with tunable properties, but it seems that, in order for such hydrogels systems to be used for CNS repair, full plastic compression of the collagen hydrogels should probably be avoided due to the tremendous increase in the hydrogel's stiffness after full compression. The stiffness that was found to be in line with the stiffness of the natural CNS environment corresponds to % level of compression up to 75%. If that is taken into consideration, the 3D compressed models could be a great alternative approach for neural engineering strategies in order to accomplish a model that resembles the natural hierarchical model of the human tissues, reaching a better regenerative potential.

Collagen-genipin injectable hydrogels on the other hand have been found to be very easy to use, while, in parallel, they are favoured by the neurosurgeons due to the less interventional therapeutic approach that can be implemented. The gels exhibit mechanical properties similar to the CNS tissue and they have previously been found to adequately support cells growth, facilitating neural regenerative processes. The genipin concentration though should probably be optimized and longer-term studies should test the degradation rate of the optimized hydrogel over time for optimal structural support that will allow adequate regeneration.

Overall, the tested hydrogel systems hold promise for CNS applications, but it is still very soon to conclude on which system is the best for clinical applications, since both hydrogel systems need to be further optimized and tuned. Longer in vitro, as well as in vivo, studies need to be conducted to check the efficiency of those systems more accurately. It seems that the clinical potential of neural engineering strategies is endless and is about to improve in the near future.

## Figures and Tables

**Table 1:** Correlation of the hydrogels' stiffness with the % compression rate.

% fluid loss	Young's modulus (kPa)
0	20.86 ± 3.20
50	40.14 ± 17.10
75	230.51 ± 41.00
99	2238 ± 776
